# A Case of Persistent Hyperglycemia: Autoimmune Link Between Hashimoto’s Thyroiditis and Latent Autoimmune Diabetes in Adults

**DOI:** 10.7759/cureus.91219

**Published:** 2025-08-29

**Authors:** Ahmed Ibrahim, Mohmed Hussien Ahmed Mohmed, Haytham Darwish, Pierre Maximus, Manal Rezzek, Mahmoud HS Daoud, Ahmad Labad

**Affiliations:** 1 Diabetes and Endocrinology, Hatta Hospital, Dubai, ARE; 2 Medicine, Gezira University, Madani, SDN; 3 Education and Nursing, Hatta Hospital, Dubai, ARE

**Keywords:** anti beta-cell autoantibodies, diabetes mellitus, hashimoto's thyroiditis, latent autoimmune diabetes in adults, type 1 diabetes mellitus, type 2 diabetes mellitus

## Abstract

Latent autoimmune diabetes in adults (LADA), also known as type 1.5 diabetes, is an autoimmune diabetes that shares genetic, immunological, and clinical features with type 1 diabetes mellitus (T1DM) and type 2 diabetes mellitus (T2DM) and is commonly misdiagnosed as type 2 diabetes. Thyroid disease in patients with LADA was estimated to be 17.7%. We are reporting a case of Hashimoto's thyroiditis (HT) in an obese patient having hyperglycemia with poor response to regular management. We reported this case as a reminder that the presence of an autoimmune condition in a patient with hyperglycemia, autoimmune diabetes, must be considered.

A 40-year-old female with a body mass index of 35.2 kg/m² complains of fatigability and exercise intolerance. Her HbA1c was 7.9; she was first diagnosed with T2DM and started on the oral hypoglycemic agent metformin. Her HbA1c increased despite her admitting that she was adherent to her medications and the dietary regimen. She reported anterior neck swelling. The thyroid ultrasound showed a heterogeneous texture of the thyroid gland. Anti-thyroid peroxidase antibody titer and anti-thyroglobulin antibody titer were high. Her anti-TSH receptor antibody titer was within the normal range. Thus, she was diagnosed with autoimmune HT and started on thyroxine. Both parents had T2DM, and she was never admitted to the hospital with ketoacidosis. Failure to control her diabetes, along with the recent diagnosis of autoimmune HT, raises the suspicion of LADA. Her anti-glutamic acid decarboxylase (GAD) antibody then came back positive, and her C-peptide level was low. She was shifted to an insulin basal and bolus regimen, and her last HbA1c was 5.7.

The case highlights the importance of considering LADA in patients with poor glycemic control despite standard T2DM therapy, especially when autoimmune diseases are present: positive anti-GAD antibodies, low C-peptide, and improved outcomes with insulin support the diagnosis. Early detection is crucial for effective management and preventing disease progression.

## Introduction

Latent autoimmune diabetes in adults (LADA) is a form of autoimmune diabetes, similar to type 1 diabetes mellitus (T1DM), which occurs due to the destruction of pancreatic beta cells, resulting in insulin insufficiency [1]. The diagnostic criteria, as outlined by the Immunology of Diabetes Society, include a minimal age of 30 years at the onset of diabetes, the presence of at least one circulating islet autoantibody, and a lack of insulin requirement for at least six months after diagnosis [2].

LADA has some similarities with type 2 diabetes mellitus (T2DM), like obesity, decreased physical activity, and other criteria of metabolic syndrome predisposing to insulin resistance; therefore, LADA is commonly misdiagnosed as T2DM [3,4]. Importantly, T1DM can present earlier with manifestations of diabetic ketoacidosis, even as the first presentation, and hyperglycemia requires prompt recognition and restricted management. However, as in our case, LADA might not present first as DKA [5,6].

Despite the similarities between LADA and T1DM, the requirement for insulin therapy may be delayed, as some beta-cells are still functioning. However, this function will decrease as the disease progresses. LADA is difficult to control through diet modification, exercise, and oral hypoglycemic agents as T2DM is managed, but early introduction of insulin in LADA is mandatory [3,7].

Hashimoto's thyroiditis (HT) is an inflammatory autoimmune disorder that can present clinically with or without a goiter. It may present with or without a goiter (enlarged thyroid) and is more prevalent in women. Common symptoms include fatigue, weight gain, cold intolerance, dry skin, constipation, and depression. Diagnosis is typically confirmed by elevated thyroid-stimulating hormone (TSH), low free thyroxine (T4), and the presence of thyroid peroxidase (TPO) antibodies [8]. HT has a higher incidence than all other autoimmune diseases, with a rate eight times higher in females than in males [8,9]. With evidence that autoimmune diseases interact with each other regarding their frequencies, time of onset, and clinical presentation, there are several studies suggesting a strong interaction between DM and HT [10]. The prevalence of thyroid disease in patients with LADA is estimated to be 17.7% [11]. Significantly, the presence of more than one beta-cell antibody is associated with a predisposition to autoimmune thyroid diseases as a comorbidity. Similarly, the presence of anti-TPO is associated with accelerated autoimmune destruction of beta-cells [12,13].

Autoimmune polyglandular syndrome, characterized by the coexistence of more than two autoimmune diseases, is commonly reported compared to the few studies regarding T2DM, and even fewer studies have reported on the coexistence of LADA and autoimmune thyroiditis [14].

We are reporting a case of developed HT in an obese patient having hyperglycemia diagnosed initially as T2DM with poor response to diet, exercise, and oral hypoglycemic agents. The coexistence of HT raised suspicion of the presence of LADA, considering her age. Therefore, we report this case as a reminder that the presence of an autoimmune condition in a patient with hyperglycemia and autoimmune diabetes must be considered so that the patient can receive appropriate management.

## Case presentation

A 40-year-old woman presented to the clinic in December 2019, complaining of persistent fatigue and exercise intolerance. She reported no polyuria, polydipsia, or unexplained weight loss. On systemic review, the patient reported no additional symptoms. She denied headaches, visual disturbances, chest pain, palpitations, or shortness of breath. There was no history of abdominal pain, nausea, vomiting, or changes in bowel habits. She reported no joint pain, muscle weakness, or skin rashes, and her menstrual cycles were regular.

Her medical history was unremarkable, with no prior diagnoses of hypertension, dyslipidemia, or cardiovascular disease. She had no history of smoking or alcohol use. Family history indicated that both of her parents had T2DM, but there was no family history of autoimmune diseases or thyroid disorders. She is a schoolteacher, not a smoker, and she does drink alcohol.

Upon physical examination, the patient looks obese, with a BMI of 35.2 kg/m². Her vital signs were within normal limits: blood pressure, 128/82 mmHg; heart rate, 76 bpm; respiratory rate, 16 breaths per minute; and temperature, 36.8°C. The systemic examination, which included cardiovascular, respiratory, abdominal, and neurological assessments, was unremarkable. There were no signs of acanthosis nigricans, skin infections, or peripheral neuropathy.

Initial blood tests revealed an HbA1c level of 7.9%, leading to a diagnosis of T2DM. She was started on metformin and followed regularly in the diabetes clinic. Despite treatment adherence, her blood glucose levels and HbA1c continued to rise. By June 2020, her HbA1c had increased to 8.6%, prompting an increase in her metformin dose and the addition of sitagliptin. She also consulted a dietitian, received dietary advice, and was encouraged to adopt lifestyle changes and lose weight.

By September 2020, her HbA1c had increased to 9.9%, despite her reported adherence to medications and dietary recommendations. In January 2021, her HbA1c rose sharply to 11.7%. At this time, she also reported symptoms of polymenorrhagia and anterior neck swelling. Blood tests revealed microcytic anemia with a hemoglobin level of 8.5 g/dL. An abdominopelvic ultrasound ruled out uterine fibroids but identified multiple ovarian cysts, consistent with polycystic ovary syndrome. Her TSH level was 8.5 mIU/L, and her free T4 level was 0.6 ng/dL. A thyroid ultrasound revealed a heterogeneous thyroid texture with increased vascularity, suggestive of inflammatory thyroiditis. Further testing revealed an elevated erythrocyte sedimentation rate (ESR) of 20 mm/h, anti-TPO antibodies of 309 IU/mL, and anti-thyroglobulin antibodies of 300 IU/mL. Anti-TSH receptor antibodies were within the normal range. Based on these findings, she was diagnosed with HT and started on thyroxine and iron supplements, and her free T4 13.5, free T3 4.2, and TSH 4.2 were controlled with thyroxine replacement.

For her diabetes management, gliclazide was introduced and titrated up to 120 mg/day. Additionally, dapagliflozin (an SGLT2 inhibitor) and dulaglutide (a GLP-1 agonist) were added to her regimen. Despite these interventions and close follow-up over six months, her HbA1c remained elevated at 11.4% in June 2021. This persistent poor glycemic control, despite adherence to multiple oral hypoglycemic agents and lifestyle modifications, raised suspicion of LADA. Although she was obese and had a strong family history of T2DM (both parents had T2DM), her concurrent diagnosis of HT and failure to respond to standard diabetes treatments supported the possibility of LADA.

**Figure 1:**
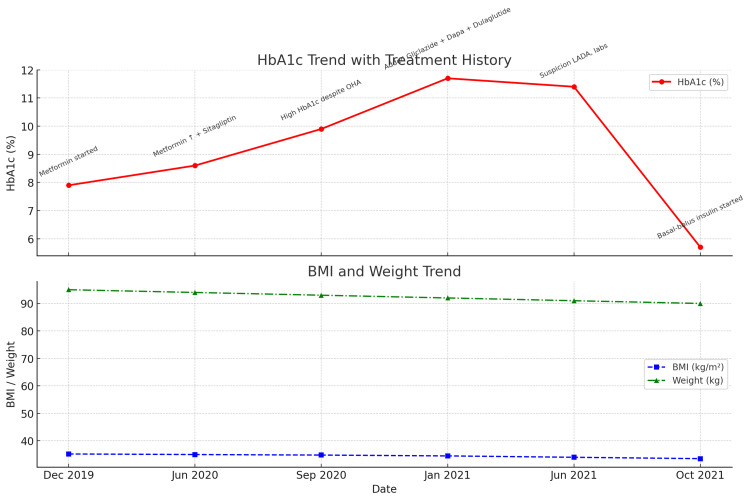
Time-series trend of HbA1c, body weight, and BMI along with the treatment history HbA1c: glycated hemoglobin, BMI: body mass index, LADA: latent autoimmune diabetes in adults

Further investigations were performed to confirm the diagnosis. Her blood ketone level was elevated at 1.6 mmol/L (normal <0.6 mmol/L), anti-GAD antibodies were positive, and her C-peptide level was very low. These findings confirmed the diagnosis of LADA. All oral hypoglycemic agents were discontinued, and she was transitioned to a basal-bolus insulin regimen. By October 2021, her HbA1c had significantly improved to 5.7%, reflecting excellent glycemic control (Figure 1, Table 1).

**Table 1:** Patient’s lab results ESR: erythrocyte sedimentation rate, TPO: thyroid peroxidase, TSH: thyroid-stimulating hormone, GAD: glutamic acid decarboxylase

Parameter	Patient’s lab results	Normal reference range
HbA1c	7.9-11.7%	<5.7% (normal), 5.7-6.4% (pre-diabetes), ≥6.5% (diabetes)
Hemoglobin	8.5 g/dL	12.0-15.5 g/dL (women)
ESR	20 mm/hr	<20 mm/hr
Anti-TPO antibodies	309 IU/mL	<34 IU/mL
Anti-thyroglobulin antibodies	300 IU/mL	<115 IU/mL
Anti-TSH receptor antibodies	Normal	<1.75 IU/L (typical reference)
Blood ketones	1.6 mmol/L	<0.6 mmol/L
Anti-GAD antibodies	Positive	Negative
C-peptide	Very low	0.8-3.1 ng/mL (fasting)

## Discussion

LADA, also known as type 1.5 diabetes, is autoimmune diabetes that shares genetic, immunological, and clinical features with T1DM and T2DM (Table 2) [15]; consequently, LADA is misdiagnosed as T2DM at a rate of 5-10% [16]. The clinical findings, like obesity in our case, in addition to hyperglycemia, and, according to international guidelines from the American Diabetes Association (ADA) [17], the classification of diabetes at diagnosis is based on clinical presentation, glycemic indices, and patient-specific risk factors such as age, obesity, and family history. In this case, the patient was initially diagnosed with T2DM in accordance with these guidelines, given her age, obese BMI, and strong family history of T2DM. However, her poor response to management with concomitant HT made autoimmune diabetes (LADA) a possible diagnosis with a good response to new therapy. Epidemiologically, Naik et al. [18] observed that LADA may account for 2% to 12% of all cases of diabetes in the adult population, providing evidence that this type of adult-onset autoimmune diabetes is relatively common.

**Table 2:** Differences and similarities between T1DM, T2DM, and LADA T1DM: type 1 diabetes mellitus, T2DM: type 2 diabetes mellitus, LADA: latent autoimmune diabetes in adults [16,19-21]

	LADA	T1DM	T2DM
Age of onset in years	30-50, but may occur at any age	Less than 30	More than 25 typically
Onset	Acute or subacute, but rarely as DKA	Acute and common DKA	Commonly subclinical
Body weight	Normal to overweight	Normal	Overweight to obese
C-peptide level	Low to normal	Low to undetectable	Normal to high
Presence of antibodies	Present	Present	Not present
Co-existence of other autoimmune	Common	Common	Less common
Progression to insulin requirement	Slow	Immediate	Slow to never

Autoimmune diabetes possesses a different presentation and response to treatment at the time of diagnosis, ranging from diabetic ketoacidosis to hyperglycemia controlled by diet modification and exercise. Consequently, individuals diagnosed with LADA may not require insulin earlier, exhibiting a wide range of phenotypes, from the presence of insulin resistance to insulin deficiency resulting from beta-cell destruction [3,7,15], which falls somewhere between T1DM and T2DM.

Our case supports the hypothesis that both autoimmunity and insulin resistance can contribute to adult-onset diabetes. The patient demonstrated high anti-GAD titers, poor glycemic response to standard therapy, and coexisting HT. These findings are consistent with reports that patients with high GAD antibody titers often exhibit greater insulin deficiency and broader autoimmunity, as indicated by the presence of additional antibodies such as TPO [22,23].

There are four types of islet autoantibodies mainly encountered in patients with LADA: anti-GAD65, zinc transporter 8 antibodies, and IA-2, with anti-GAD65 being the most sensitive [24]. The most important diagnostic markers of LADA are C-peptide levels and autoantibodies against islet antigens [25]. Importantly, any patient suspected of having LADA must measure C-peptide, which may be at a lower level, as in our case; however, the presence of anti-GAD65 supports the diagnosis [26]. Furthermore, patients having a higher titer of anti-GAD65 are likely to have a T1DM-like clinical picture due to more islet cell destruction [27]. Contrarily, patients with a lower titer of anti-GAD65 develop a T2DM-like clinical picture [28], similar to our case, which was initially misdiagnosed as T2DM and showed a poor response to management.

HT is an autoimmune inflammatory disease that occurs at higher rates in females than in males. It occurs due to pathological infiltration of lymphocytes in the thyroid gland, and 95% of cases test positive for anti-TPO and 60-80% for TG. HT can present with or without goiter and reduced echogenicity ultrasound findings [8]. For instance, the detection of islet cell autoantibodies and low-to-normal C-peptide levels is characteristic of LADA, distinguishing it from T2DM [1]. Additionally, the co-occurrence of autoimmune thyroid disease, particularly HT (characterized by elevated TSH, low free T4, and positive TPO antibodies), is frequently reported in patients with LADA due to shared autoimmune etiology [2]. Our case presented with goiter and tested positive for anti-TPO and TG, with ultrasound findings reporting a heterogeneous texture of the thyroid gland, multiple echogenic septae, and increased vascularity. Consequently, our patient was diagnosed with HT, and the poor response of our patient to management as T2DM and the presence of autoimmune thyroiditis raised the suspicion of concomitant autoimmune diabetes, as many studies suggested that a personal or family history of the autoimmune condition in a patient with hyperglycemia alerts the presence of LADA or T1DM [29].

There is a paucity of data regarding the optimal initial treatment of LADA; however, recent studies have suggested that the use of dipeptidyl peptidase 4 inhibitors may be beneficial and can delay the destruction of beta-cells. Further randomized trials may be necessary to confirm this finding. Additionally, there is a lack of data regarding the use of metformin in LADA, and evidence suggests that sulfonylureas are discouraged due to the rapid deterioration of beta-cell function. There is evidence that insulin therapy should be initiated to treat patients with LADA to control blood sugar [3,30], supporting the use of insulin in our case, given the good response, as evidenced by her last HbA1C of 5.7, despite the failure of oral hypoglycemic medications to control her blood sugar. Although the patient’s bleeding-related anemia may have caused the HbA1c to underestimate glycemic control, indicating that the true degree of metabolic disturbance was likely greater than the HbA1c value suggested.

Similar to reports of three elderly patients initially treated as T2DM but later confirmed to have LADA, our patient showed a poor response to oral therapy and required insulin after antibody testing. Although younger and obese, she followed a comparable diagnostic pathway, reinforcing that LADA can present across various age groups and body types and warrants early antibody testing when glycemic control is suboptimal [31].

In a case series conducted in 2025, 10 patients were diagnosed as LADA, of whom 20% had HT. Several were initially treated as T2DM and later required insulin, underscoring the T2DM-like onset with coexisting thyroid autoimmunity seen in our patient [32].

Our case highlights that the presence of autoimmune disease in a patient diagnosed with T2DM mellitus with poor response to appropriate management must raise the suspicion of adult-onset autoimmune diabetes so that the patient can receive adequate treatment.

## Conclusions

The presented case highlights the importance of considering LADA in patients with poor glycemic control despite adherence to T2DM management, particularly when autoimmune conditions such as HT are present. Positive anti-GAD antibodies and low C-peptide levels confirmed the diagnosis, and insulin therapy significantly improved glycemic outcomes. Early recognition of LADA is crucial for guiding appropriate treatment and preventing disease progression.
